# Form, Fabric, and Function of a Flagellum-Associated Cytoskeletal Structure

**DOI:** 10.3390/cells4040726

**Published:** 2015-11-03

**Authors:** Brooke Morriswood

**Affiliations:** Department of Cell and Developmental Biology, Biocenter, University of Würzburg, Am Hubland, D-97074, Würzburg, Germany; E-Mail: brooke.morriswood@uni-wuerzburg.de; Tel.: +49-931-31-83556

**Keywords:** *Trypanosoma brucei*, cytoskeleton, TbMORN1, MORN-repeat, BioID

## Abstract

*Trypanosoma brucei* is a uniflagellated protist and the causative agent of African trypanosomiasis, a neglected tropical disease. The single flagellum of *T. brucei* is essential to a number of cellular processes such as motility, and has been a longstanding focus of scientific enquiry. A number of cytoskeletal structures are associated with the flagellum in *T. brucei*, and one such structure—a multiprotein complex containing the repeat motif protein TbMORN1—is the focus of this review. The TbMORN1-containing complex, which was discovered less than ten years ago, is essential for the viability of the mammalian-infective form of *T. brucei.* The complex has an unusual asymmetric morphology, and is coiled around the flagellum to form a hook shape. Proteomic analysis using the proximity-dependent biotin identification (BioID) technique has elucidated a number of its components. Recent work has uncovered a role for TbMORN1 in facilitating protein entry into the cell, thus providing a link between the cytoskeleton and the endomembrane system. This review summarises the extant data on the complex, highlights the outstanding questions for future enquiry, and provides speculation as to its possible role in a size-exclusion mechanism for regulating protein entry. The review additionally clarifies the nomenclature associated with this topic, and proposes the adoption of the term “hook complex” to replace the former name “bilobe” to describe the complex.

## 1. Introduction

The trypanosomatids are a group of uniflagellated protists belonging to the class Kinetoplastida in the eukaryotic super-group Excavata [1]. They are obligate parasites. As objects of scientific study, there are at least four reasons why trypanosomatids are of outstanding interest: (i) as pathogens and the causative agents of several neglected tropical diseases, they are responsible for a significant health and economic burden on some of the poorest human communities [2]; (ii) as members of the super-group Excavata, they are evolutionarily distant to most studied organisms and provide an invaluable reference point in the context of evolutionary cell biology, with both conserved and novel features [3,4]; (iii) they exhibit (like many unicellular organisms) certain cellular features in a simplified and streamlined form, making them good model systems for a number of fundamental questions in eukaryotic cell biology; (iv) they are capable of manipulating their hosts (for example, by causing changes to the circadian rhythm), making them effective tools for studying host physiology [5,6].

**Figure 1:**
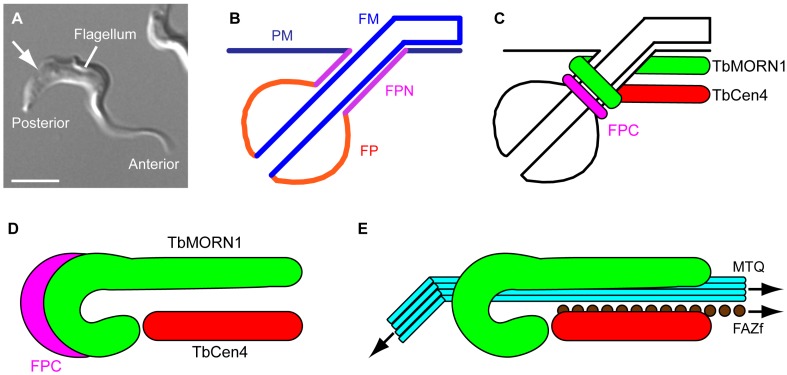
Membrane subdomains and the morphology of the TbMORN1-containing complex. (**A**) A BSF *T. brucei* cell. The anterior and posterior ends of the cell, and the single flagellum, are labelled. The approximate point where the flagellum enters the cell body is indicated (arrow). Scale bar, 5 µm; (**B**) Schematic showing membrane subdomains in the region of the flagellar pocket. The plasma membrane (PM), flagellar pocket neck (FPN), flagellar pocket (FP), and flagellar membrane (FM) are composed of one continuous lipid bilayer but constitute four distinct morphological subdomains; (**C**) Schematic showing the position of the flagellar pocket collar (FPC) and the inferred positions of the multiprotein complexes containing TbMORN1 and TbCentrin4 in the region of the FPN; (**D**) Schematic showing the FPC, TbMORN1, and TbCentrin4 in a “top down” orientation. Colour scheme same as in part C; (**E**) Schematic showing the position of TbMORN1 and TbCentrin4 relative to the cytoplasmic components of the flagellum attachment zone (FAZ). These FAZ components are termed the microtubule quartet (MTQ) and FAZ filament (FAZf). For clarity, the flagellum and FPC have been omitted.

Of the various species of trypanosomatids, the African trypanosome (*Trypanosoma brucei*) is the best characterised in a laboratory setting. Its life cycle involves transitions between the tsetse fly (the definitive host) and mammals (intermediate host), with several developmental stages being manifested [7,8,9]. Of these developmental stages, the procyclic form (PCF, found in the tsetse fly) and bloodstream forms (BSF, found in mammalian hosts) have been studied the most intensively. Both PCFs and BSFs show trypomastigote morphology, with the single flagellum emerging from the cell posterior and then adhering lengthwise to the cell body, with the tip extending beyond the anterior end of the cell [10]. The cell has a fusiform shape, with a wider posterior end and a narrower anterior (Figure 1A).

Below the plasma membrane is a corset of microtubules that encloses the intracellular organelles and maintains cell shape [11]. The microtubules are orientated with their minus ends at the cell anterior end, and the plus ends clustered at the posterior cell pole [12]. Near the posterior end of the cell is a small flask-shaped invagination of the plasma membrane—the flagellar pocket (FP) (Figure 1B). The FP is the sole site of endo- and exocytosis [13,14]. At the base of the FP, on the intracellular side, is the single basal body that nucleates the flagellum axoneme [15]. A probasal body is positioned perpendicular to it. During cell replication, the probasal body matures into the new basal body, which then nucleates the new flagellum.

The flagellum, enclosed in the flagellar membrane, transits the FP and then follows a helical path around the exterior of the cell body, describing an approximately 180-degree turn by the time it reaches the anterior end of the cell [16]. The flagellum is motile and beats primarily in a tip-to-base manner [17]. Although the flagellum beat is planar, it results in a helical waveform owing to the coiled path of the flagellum around the cell body, and thereby the cell's characteristic corkscrew-like swimming motion (trupanon is Greek for “borer”) [16].

A requirement for this transmission of the flagellum beat to the cell body is adhesion of the flagellum to the cell, and the structure responsible for this is the flagellum attachment zone (FAZ) [18,19,20]. The FAZ is composed of three main elements: (i) fibres within the flagellum that link the axoneme and paraflagellar rod to the flagellar membrane; (ii) inter-membrane connections that link the flagellar membrane and plasma membrane; (iii) two polymers within the cell body—the microtubule quartet and the FAZ filament [19,21,22,23]. The microtubule quartet is a specialised quartet of microtubules which are presumed to have the opposite polarity to those of the corset [12]. They are nucleated between the basal body and probasal body, diagonally wrap around the FP on its cytoplasmic face, and then run parallel to the flagellum to the anterior tip of the cell [24]. A subdomain of the ER is interdigitated between the microtubules of the microtubule quartet [25]. The FAZ filament is a cytoskeletal thread, which runs alongside and to the right of the microtubule quartet (when viewed from the cell posterior end) and has a “string of beads” appearance when imaged by electron microscopy [24]. The posterior end of the FAZ filament initiates at the neck of the FP.

The flagellar pocket neck—along with the FP and flagellar membrane—constitutes a third distinct morphological subdomain of the plasma membrane (Figure 1B) [24,26]. Unlike the rest of the FP, where membrane balloons out in a spherical manner, the flagellar pocket neck membrane domain is cylindrical and tightly apposed with the flagellar membrane. Freeze-fracture analysis of integral membrane protein distributions indicate that its composition is similar to the surface plasma membrane [27].

At the lower end of the flagellar pocket neck, atop the bulge of the FP, is an electron-dense cytoskeletal structure called the flagellar pocket collar (FPC) (Figure 1C). It is horseshoe- or sigma-shaped, with the ends positioned to allow passage of the microtubule quartet [24]. To date, only one component of the FPC has been characterised—TbBILBO1 [28,29,30,31]. 

In addition to the FPC, at least two other multiprotein cytoskeletal complexes are present in the flagellar pocket neck region. One is defined by the protein TbCentrin4 [32,33]. The other contains the repeat-motif protein TbMORN1, and is the subject of this review (Figure 1C) [34]. The form (morphology), fabric (composition) and function of the TbMORN1-containing complex will be considered.

## 2. Form

### 2.1. The Shape of Things

The TbMORN1 protein (40 kDa) has a highly repetitive primary structure, consisting of fifteen consecutive 23-amino acid MORN (membrane occupation and recognition nexus) repeats [34,35]. The macromolecular TbMORN1-containing complex is ~2 µm long. The posterior end of this complex is tightly curled around the cytoplasmic face of the flagellar pocket neck, producing an overall fishhook-shaped morphology consisting of a posterior “bend” plus an anterior “shank” (Figure 1D) [36]. For simplicity, and to clearly differentiate it from statements referring to the TbMORN1 protein, the TbMORN1-containing complex will be referred to as the “hook complex” from here on. Fluorescence recovery after photobleaching (FRAP) analysis of GFP-tagged TbMORN1 has shown that its turnover on the hook complex is very low [36]. This low turnover is to be expected of a stably-incorporated structural component of the cytoskeleton.

Like the FPC, TbMORN1 shows a tight association with the flagellum. The flagellar axoneme, basal body, and FAZ can be purified using high salt conditions in order to depolymerise the cytoskeletal microtubule corset [37]. Under such conditions, TbMORN1 co-purifies with the flagellum [34]. These preparations of isolated flagella, with the FPC and TbMORN1 still attached, have been the basis of morphological study using a combination of immunofluorescence microscopy and electron microscopy (EM) [36].

Analysis of the preparations confirmed that the TbMORN1 anterior shank is positioned asymmetrically on one side of flagellum. It is adjacent to the microtubule quartet and may partially overlap with it [36]. Running parallel to the anterior shank part of TbMORN1 is the complex defined by TbCentrin4, with a rod-shaped morphology. This complex lies alongside the FAZ filament (Figure 1E) [36]. The TbCentrin4-containing complex will be referred to as the “centrin arm” from here on. The position of the centrin arm roughly corresponds to that described for the “neck microtubule”, a single microtubule entirely contained within the flagellar pocket neck region [24]. Whether the centrin arm and the neck microtubule are one and the same structure remains to be definitively established.

The posterior bend part of TbMORN1 overlaps with the FPC. However, immunofluorescence images taken when the flagellum was lying at an oblique angle revealed that the overlap between TbMORN1 and TbBILBO1 is only partial. This is consistent with a model in which TbMORN1 is positioned on top of the FPC, an interpretation further supported by immuno-EM data [36].

It remains something of a puzzle why all three complexes co-purify with the flagellar axoneme. There are no fewer than two lipid bilayers (the flagellar membrane and flagellar pocket neck) separating them, yet the association is tight enough to withstand detergent and high salt treatment. It is not clear if this is due to the presence of unidentified transmembrane physical connections between the structures, or if the co-purification might instead reflect a tight association with either the microtubule quartet and/or the FAZ filament.

Another question in the morphological studies concerns visualisation. The electron-dense FPC is readily visible without labelling, and indeed the same is true for the centrin arm, but the hook complex is only clearly visible at an EM level using immuno-labelling. This fact may explain why the hook complex was not discovered earlier, given the extensive and high-quality extant literature on trypanosome ultrastructure. A key goal for future studies then is the establishment of fixation, staining and contrast conditions that render the hook complex visible.

Conceptually, the main outstanding morphological question is probably whether these three multiprotein complexes—the FPC, hook complex, centrin arm—are a single entity with discrete subdomains, or (two? three?) distinct structures inhabiting separate regions. Immuno-EM with anti-TbMORN1 and anti-TbCentrin4 antibodies showed that although the majority of each protein is found on one side or the other of the FAZ filament, the distribution is not binary [36]. This suggests that small quantities of each protein are present in both complexes and that together the hook complex and centrin arm may comprise a single hairpin-shaped entity. Additional high-resolution analysis (for example, using super-resolution light microscopy) would address this issue. It will also be important to deplete or overexpress individual components of each of the complexes and assess the impact of this perturbation on all three structures. Depletion of TbMORN1 has been shown not to affect the FPC in BSF cells [38]. However, a more systematic evaluation of the interdependencies of the complexes has not yet been carried out.

Finally, MORN1 has not yet been characterised in other trypanosomatids, despite the existence of clear orthologues in *Trypanosoma cruzi* and *Leishmania* species (*i.e.*, proteins of 358 amino acids composed of 15 MORN repeats only). The *T. cruzi* orthologue (TcCLB.511729.60) has 84% sequence identity with TbMORN1; orthologues in *Leishmania* species have around 76%–78% sequence identity. Given the large differences in cellular form between these species and *T. brucei*, it will be interesting to determine whether the MORN1 orthologues are exhibiting similar morphologies.

### 2.2. Replication Mechanism

Another major area of enquiry concerns how the hook complex is replicated. Most cytoskeletal components are assembled *de novo*, and preventing the manufacture of new protein using RNAi generally results in an unaffected old/parental structure, and a failure of assembly of a new/daughter structure. In contrast to other cytoskeletal structures however, it seems that during replication of the hook complex, the old/parental structure makes a physical contribution to the composition of the new/daughter structure. Despite FRAP indicating that the turnover of TbMORN1 molecules on the hook complex is negligible, when TbMORN1 is depleted by RNAi both the old and the new structures appear to be affected [38].

Replication of the hook complex appears to be primarily coordinated by the mitotic kinase TbPLK [39,40,41]. TbPLK appears to sequentially interact with and possibly also phosphorylate a number of distinct cytoskeletal proteins in the basal body, hook complex, centrin arm, and FAZ [42]. Currently, it is not known if other mitotic kinases are involved, and a number of other questions remain. Determining the mechanism by which TbPLK orchestrates replication, and the order of assembly of new components in each of the structures, are likely to be key goals for future research.

A further question concerns how new material reaches the replicating structures. At both an immunofluorescence microscopy and an immuno-EM level, tendrillar projections that connect the replicating hook complex to the basal body can be observed [36,40]. The earliest known event in the trypanosome cell cycle is probasal body maturation, shortly followed by outgrowth of the new microtubule quartet [15]. This suggests that the tendrillar projections may represent newly-synthesised material being trafficked along the old and new microtubule quartets. Consistent with this idea, FRAP data has shown a higher turnover of material on the tendrillar projections [36]. However, no immunofluorescence overlap has been observed between anti-tubulin and anti-TbMORN1 antibodies on the first tendrillar projection to form [36]. Whether the structure is in fact the microtubule quartet but so coated with material that it precludes antibody access remains open to question.

In a broader context, it is not known if the hook complex assists in the replication of other cellular structures, in particular the FP. The FPC has already been shown to be essential for FP replication—when TbBILBO1 is depleted using RNAi the basal body and flagellum are efficiently duplicated, but no new FP is formed [28]. TbBILBO1 RNAi is lethal in PCF cells, while TbMORN1 RNAi is not, which may suggest that TbMORN1 is not required [28,34]. The full choreography of FP biogenesis has only recently begun to be deciphered however, and it is clearly a complex and highly-coordinated process. In particular, it has been shown that a membrane profile (the “ridge”) seems to invade the FP and presumably divides the volume into two, while the new basal body and associated new microtubule quartet undergo an anti-clockwise rotation around the old flagellum [15]. It is tempting to speculate that the tendrillar projections mentioned in the paragraph above could be a cytoskeletal scaffold that transiently forms to facilitate these processes. Resolving these questions is likely to be a fruitful area of enquiry.

Finally, is there any relationship to *Toxoplasma gondii* MORN1? MORN-repeats are found (singly or in multiple copies) in proteins from all domains of life, which complicates the identification of direct TbMORN1 orthologues—*i.e.*, proteins containing 15 MORN repeats and no other domains. To date, the only characterised TbMORN1 orthologue is in *Toxoplasma gondii* (phylum Apicomplexa) with 57% sequence identity [43,44]. Extensive work has shown that this protein is involved in cellular replication at an early stage of the cell cycle [45,46,47,48,49]. The unusual replication mechanism of the Apicomplexa involves the formation of daughter cells within the cytoplasm of the mother, a process that requires the progressive enlargement of a double-membrane structure that ultimately encases the immature daughter cell. *Toxoplasma gondii* MORN1 localises to a ring-shaped complex present at the ends of this double membrane. Topologically, this situation is reminiscent of the localisation of TbMORN1 in *T. brucei*, with the invagination of the FP resulting in a continuous double-membrane subdomain of the plasma membrane (Figure 1B). As such, it may be the case that a similar replication process might be in operation.

## 3. Fabric

### Composition of the Hook Complex

When dealing with an uncharacterised structure, the next goal after describing its morphology is an inventory of its components. This is harder for cytoskeletal structures than for cytoplasmic complexes however, as they partition into the detergent-insoluble fraction. As such, affinity-purification techniques that rely on solubilisation in non-ionic detergent (for example, co-immunoprecipitations and TAP tagging) are non-viable. A new technique that shows great promise in addressing this problem is proximity-dependent biotin identification (BioID) [50]. The hook complex was one of the first cellular structures to be interrogated using BioID, and its application in this case remains a good example of the technique’s strengths [51].

BioID involves tagging a protein of interest with a modified version of the bacterial biotin ligase BirA (BirA*) [52]. BirA normally catalyses the conjugation of an adenine nucleotide to biotin in order to generate the highly reactive intermediate biotinoyl-5′-AMP [53]. This intermediate is retained within the enzyme's active site until a protein with the correct recognition sequence is located. BirA then conjugates the biotin to an acceptor lysine residue within the recognition sequence. BirA has previously been exploited by introducing the recognition sequence within a protein of interest, in order to produce targeted biotinylation [54].

**Figure 2:**
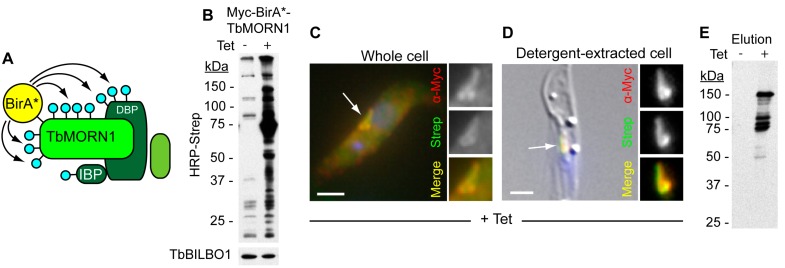
Use of BioID to identify components of the hook complex. (**A**) Schematic showing the BioID principle. TbMORN1, tagged with the BirA* module, is depicted in a complex with a direct binding partner (DBP) and an indirect binding partner (IBP). Biotinylation is represented by blue lollipops. Proteins not in proximity to TbMORN1 are not biotinylated; (**B**) Immunoblots of whole cell lysates taken from cells inducibly expressing myc-BirA*-TbMORN1. Expression is switched on in the presence of tetracycline (Tet). Biotinylated proteins were detected using HRP-conjugated streptavidin. In +Tet conditions, a large increase in the number and amount of biotinylated proteins was observed. Immunoblotting with anti-TbBILBO1 antibodies confirmed that the protein concentration in the -Tet and +Tet lysates was similar (lower panel); (**C**) Immunofluorescence analysis of a cell inducibly expressing myc-BirA*-TbMORN1 in the presence of excess biotin. Anti-myc antibodies were used to detect the tagged TbMORN1 isoform; biotinylated proteins were detected with fluorophore-conjugated streptavidin. A good overlap between the two labels was observed in the region of the complex; (**D**) Immunofluorescence analysis of a detergent-extracted cell. Labelling was the same as shown in panel C. Scale bars C-D, 2 µm; (**E**) Purification of putative cytoskeletal binding partners and near neighbours of TbMORN1. Elutions were probed with HRP-conjugated streptavidin. Excellent signal-to-noise was observed compared to control (-Tet) conditions. The data shown in this figure recapitulate previously-published work [51].

BirA* carries a single point mutation that dramatically reduces its affinity for the biotinoyl-5′-AMP product [52]. Consequently, instead of the product being retained in the enzyme's active site until a recognition sequence is located, the biotinoyl-5'-AMP is released. Owing to its high reactivity, it will conjugate to the first lysine residue it encounters. Due to the high concentration of proteins inside the cell, the molecule does not have to diffuse very far in order to encounter an acceptor lysine [50,55]. As such, a protein tagged with the BirA* module will—in the presence of excess biotin—emit a continuous stream of biotinoyl-5′-AMP moieties. These will conjugate to acceptor lysines in the vicinity of the BirA* module in an unbiased fashion (Figure 2A).

This results in a proximity-dependent biotinylation, under native conditions, of all proteins in the immediate area (~10 nm) of the BirA*-tagged protein [55]. The cells can then be lysed and the biotinylated proteins affinity-purified using streptavidin. The strength of the biotin-streptavidin interaction means that far harsher lysis conditions can be used than would normally be the case for a biochemical purification—sufficient to disassemble cytoskeletal complexes. Purified proteins can then be identified using mass spectrometry. The pattern of biotinylation thus preserves spatial information independently of the lysis conditions (a necessary caveat being that a hit indicates spatial proximity but not necessarily direct interaction).

To analyse the composition of the hook complex using BioID, BirA*-tagged TbMORN1 was inducibly expressed in *T. brucei*. The generation of stable cell lines that constitutively or inducibly express ectopic protein is trivial in trypanosomes [56]. The tagged protein targeted correctly to the complex and no adverse effects in terms of cell toxicity were observed [51]. The level of endogenous biotinylation in trypanosomes is relatively low, and in addition the cells have no difficulty in taking up biotin. When expressed in the presence of excess biotin, there was a pronounced increase in both the number of biotinylated proteins, and the total amount of biotinylation relative to uninduced controls (Figure 2B). Fluorescence microscopy using fluorophore-conjugated streptavidin showed that the pattern of biotinylation matched the distribution of the BirA*-tagged TbMORN1 (Figure 2C). In detergent-extracted cells, the overlap and signal-to-noise appeared excellent (Figure 2D). Cytoplasmic proteins were then removed by detergent extraction, and the cytoskeleton solubilised using 4% SDS. Biotinylated proteins were captured using streptavidin-coated magnetic beads.

Analysing a small fraction of the eluate revealed the presence of a number of proteins at defined molecular masses (Figure 2E). The remaining proteins were directly removed from the beads using trypsin and tryptic peptides were analysed by mass spectrometry. Good candidates were readily identifiable as being highly enriched when BirA*-TbMORN1 was expressed but absent from controls. Screening ten of the most promising candidates revealed that nine localised to either the hook complex, the centrin arm, or the FAZ—a true positive rate of 90% (Table 1) [51]. Another of the top hits, besides TbMORN1 itself (the tagged protein will obviously exhibit the highest level of biotinylation of all), was the protein TbLRRP1. TbLRRP1 had previously been shown to colocalise with TbMORN1 in a proteomic screen but was the only protein known to do so prior to the use of BioID [57]. A clear advantage for the application of BioID was the fact that TbMORN1 is present in a stable cytoskeletal complex of discrete size.

Also of note is that all of the proteins identified are found exclusively in trypanosomes or trypanosomatids, with no orthologues yet documented outside the Excavata. This is by no means however a complete inventory of components. The spatial arrangement of molecules around TbMORN1 may preclude biotinylation of some neighbour proteins, and not all proteins can be efficiently identified by mass spectrometry. For example, the protein TBCCD1 has been subsequently identified as having a strongly overlapping distribution with TbMORN1 but was not present in the BioID dataset [58]. Similarly, neither TbBILBO1 nor TbCentrin4 were represented in the BioID dataset, despite their close spatial proximity to TbMORN1. Nonetheless, a tantalising possibility is that it should be possible to tag each of these newly-identified components with BirA* and repeat the process, allowing a “walk” through the structure. The “proximity map” so assembled might be a useful approximation of the arrangement of proteins in the complex as a whole. It is important to note that in such an approach, signal strength will be determined not only by proximity to the BirA*-tagged protein but also by lysine content, but the application of statistical mapping techniques should still be feasible.

There is obviously also a need to complement the BioID approach with other proteomic techniques. Sonication of preparations of isolated flagella is emerging as a powerful technique for immuno-isolating small fragments of the cytoskeleton, and has been recently employed to identify novel components of the FAZ [19]. A recent screen for TbPLK binding partners that combined BioID and phosphoproteomics independently verified the annotation of four of the known hook complex components, as well as identifying four other putative components [42]. It is not yet clear whether these four novel components are associated with the hook complex or the centrin arm however.

The current inventory of hook complex components is shown in Table 1. Only four of these characterised components appear to be common to all trypanosomatids, and one (Tb927.4.3120) is found only in the clade containing *T. brucei*. Only TBCCD1 contains a recognised domain (in the sense of a folded module of protein tertiary structure with a predicted catalytic or binding function), and predicted coiled-coils regions are present in it and a further four proteins. The protein Tb927.10.850 encodes five predicted WD40 repeats, TbLRRP1 encodes seven predicted leucine-rich repeats, and TbMORN1 contains fifteen MORN repeats. Three of the proteins encode no predicted structural repeats or domains whatsoever.

**Table 1:** Components of the hook complex.

Gene Code, Name	Size (kDa)	Tritryp Presence	Domains (Including CC)	Repeat Motifs	References
Tb927.6.4670 (TbMORN1)	41	Tb, Tc, L	-	15 × MORN	[34,36,38,51]
Tb927.4.3120	101	Tb	-	-	[51]
Tb927.7.7000	175	Tb, Tc	-	-	[51]
Tb927.8.3010	82	Tb, Tc	3 × CC	-	[51]
Tb927.10.850	168	Tb, Tc	7 × CC	5 × WD40	[42,51]
Tb927.10.3010	133	Tb, Tc	2 × CC	-	[42,51]
Tb927.10.8820	85	Tb, Tc, L	-	-	[42,51]
Tb927.11.2440 (TBCCD1)	59	Tb, Tc, L	TBCC-like, 1 × CC	-	[58]
Tb927.11.8950 (TbLRRP1)	79	Tb, Tc, L	1 × CC	7 × LRR	[42,57,59,60]

Known protein components of the hook complex are listed. “Tritryp presence” denotes whether a protein is encoded in the genomes of the related pathogenic trypanosomatids *Trypanosoma cruzi* (Tc) and *Leishmania* species (L) as well as *T. brucei* (Tb). “Domains” includes predicted coiled-coils (CC); TBCC, tubulin folding cofactor C; LRR, leucine-rich repeat. Four other candidates have recently been identified, although it is not yet clear if they are components of the hook complex or the centrin arm [42]. They are Tb927.1.4400, Tb927.4.4180, Tb927.5.2500, Tb11.01.2730.

Besides TbMORN1, only two of the components have been the subject of detailed characterisation at a cell biology level—TBCCD1 and TbLRRP1 [57,58,59,60]. Depletion of TBCCD1 in PCF *T. brucei* was found to result in a decreased presence of TbMORN1 at the complex, suggesting that it lies upstream of TbMORN1 in the assembly hierarchy and may be required for stable localisation [58]. Despite the presence of a TBCC-like domain (normally associated with the regulation of microtubule dynamics) in TBCCD1, several key catalytic residues are missing and it is not clear if the domain is functional. TBCCD1 was also shown to be required for the efficient segregation of duplicated kinetoplasts in replicating cells, but its function during interphase remains undetermined [58].

Depletion of TbLRRP1 in PCF cells also resulted in a kinetoplast segregation defect, and the protein was additionally implicated in the replication of the basal body, FPC, FAZ, centrin arm, and Golgi [57]. As with TBCCD1, the actual function of TbLRRP1 in interphase cells—as opposed to its requirement for the faithful replication of cellular structures—has not yet been clearly defined, although it has recently been suggested that TbLRRP1 can form a transient complex with a Ran-binding protein [59]. Further analysis of this interaction, and determination of how it contributes to TbLRRP1’s many proposed functions, will be extremely interesting.

While it is likely that a further delineation of components will feed back into an improved appreciation of the complex’s morphology, the lack of structural similarities between the known components of the hook complex makes it hard to make predictions as to their likely functions or that of the complex as a whole. Indeed, clarification of the function of the hook complex is undoubtedly the most pressing issue for future research.

## 4. Function

### 4.1. History and Semantics

The function of the hook complex is a source of some controversy, and relates back to its discovery and earlier work done on the replication of the trypanosome Golgi. Work in PCFs has established that the new Golgi assembles adjacent to the old one [61,62]. In mammalian cells, the Golgi is positioned next to the centrosome, the main microtubule organising centre of the cell [63]. Centrins are conserved components of microtubule organising centres across all eukaryotes, and the monoclonal antibody 20H5 has been shown to bind to centrins across many taxa [64]. Labelling PCF trypanosomes with 20H5 identified a structure in the region corresponding to the location of the hook complex and centrin arm [65]. The images taken at the time suggested it had a morphology similar to a bowling pin, and it was later named the “bilobe” (sometimes spelled “bi-lobe”). *T. brucei* expresses five different centrin isoforms, and screening epitope-tagged constructs showed that TbCentrin2 and TbCentrin4 were present at the “bilobe” [65]. Both were also present at the basal body and probasal body [65,66]. By comparing the effects of depleting TbCentrin1 (present only at the basal body and probasal body) and TbCentrin2 (present additionally at the “bilobe”) in PCF cells, it was proposed that the “bilobe” might be involved in Golgi biogenesis [65]. This is an attractive idea and the conclusion, though tentative, has been widely repeated as fact.

However, in BSF cells, the distance between the FP and the nucleus is much greater than in PCFs [67]. In these cells, the “bilobe” is now several micrometres away from the Golgi, making it hard to see how the same mechanism could be in operation [68,69].

Given too that TbCentrin2 (and TbCentrin4) are also present at the basal body and probasal body, and therefore involved in the very earliest stages of cell cycle progression in *T. brucei*, the conclusion merits some caution. The maturation of the probasal body is the earliest documented event in the cell cycle, and loss of a key basal body component is likely to have severe and pleiotropic downstream effects. What was needed—both to verify the proposed function and determine the mechanism—was a protein that localised exclusively to the “bilobe”.

TbMORN1 was thought to be that protein. When it was first characterised, and in all subsequent literature to date, it has been referred to as a marker protein for the “bilobe” [34]. However, depletion of TbMORN1 in PCF *T. brucei* does not result in any defects in Golgi replication (the author’s unpublished data).

Consequently, the only published work that suggests and supports a direct functional link between the “bilobe” and the Golgi is in the original TbCentrin2 paper [65]. Subsequent functional analysis of the trypanosome centrins has focused instead on their roles in mediating the replication (duplication and/or segregation) of a number of cellular structures and the effects of their depletion on cell cycle progression [32,33,66,70,71]. However, not all the published data are in agreement and a complete analysis of the literature would be beyond the scope of this review.

Based on these caveats, and the improved awareness of the relative dispositions of TbMORN1 and TbCentrin4 that has come from morphological studies, it seems timely to revise the usage of the word “bilobe”. “Bilobe” has been used as a catch-all term to describe a single structure containing TbMORN1, TbCentrin2, TbCentrin4 (and proteins partially or wholly overlapping with any of these components at the flagellar pocket neck). As TbMORN1 and TbCentrin4 may in fact be on separate structures (Figure 1), and given the lack of additional validation of a direct functional link to the Golgi, I would propose that the use of the term “bilobe” be discontinued.

Instead, it appears preferable to use—as here—the term “hook complex” to describe the multiprotein complex containing TbMORN1, and “centrin arm” for the rod-shaped complex containing TbCentrin4. The terms “hook complex” and “centrin arm” are more morphologically accurate, reflecting the advances in imaging made since the discovery of the “bilobe”, and are not burdened by preconceptions as to the cellular function(s) of the complexes they describe.

### 4.2. Function of TbMORN1

The function of TbMORN1, and, by extension, that of the hook complex, therefore remains an open question. Depletion of TbMORN1 by RNAi in PCFs produced a moderate growth defect but no dramatic morphological changes in the cells [34]. There appears also to be no requirement for TbMORN1 in order to achieve faithful replication of the centrin arm, FPC, FAZ, flagellum or basal body [34] (and the author’s unpublished data). The effects of TbMORN1 depletion on other components of the hook complex such as TbLRRP1 have not yet been analysed. Given that semi-quantitative immunoblotting indicated depletion by RNAi was 80% in PCF TbMORN1 RNAi cells, this means that 20% of the protein is still remaining. The generation and analysis of a conditional knockout (or even a complete knockout) is likely to be more informative.

**Figure 3:**
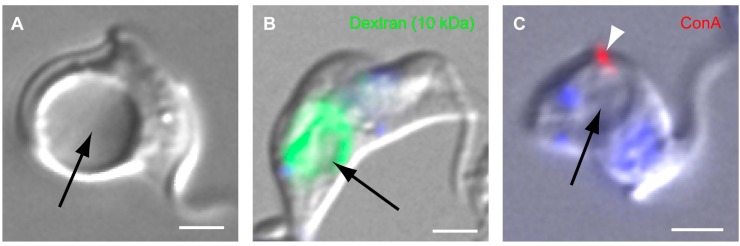
Phenotypic effects of TbMORN1 depletion in BSF cells. (**A**) A BSF cell depleted of TbMORN1 by RNAi. Image taken 14 h post-induction. The FP has become grossly enlarged (arrow); (**B**) Flourophore-conjugated dextran (10 kDa) accumulates in the enlarged FP (arrow) of TbMORN1-depleted cells; (**C**) Fluorophore-conjugated concanavalin A (ConA, arrowhead) does not accumulate in the enlarged FP (arrow). Scale bars A-C, 2 µm. The data recapitulate published work [38].

More illuminating data have recently come from analysis of TbMORN1 depletion in BSFs, which is rapidly lethal. The kinetics of TbMORN1 protein loss are roughly the same in BSFs as in PCFs [34]. Depletion of TbMORN1 in BSFs is however accompanied by a progressive enlargement of the FP, indicative of an imbalance in the endomembrane system (Figure 3A).

Enlarged FPs (a “BigEye” phenotype) are usually associated with a defect in endocytosis [72]. Consistent with this, the fluid phase marker dextran (10 kDa) accumulates inside the enlarged FP (Figure 3B) [38]. Surprisingly, however, the lectin concanavalin A (ConA) does not do so (Figure 3C) [38]. Instead, it accumulates in a single bright spot that appears to be on the cell surface. This effect is not observed in analysis of the clathrin “BigEye” phenotype, where ConA accumulates inside the enlarged FP in the same manner as dextran [72]. As ConA is a lectin, whereas dextran is a marker for fluid phase traffic, it may be the case that these different modes of uptake are responsible for the phenotype in TbMORN1-depleted cells.

However, at least one piece of evidence suggests that the mode of uptake is not the cause of the discrepancy. The protein BSA also traffics in the fluid phase but that too shows a decreased capability to access the FP in TbMORN1-depleted cells [38]. An alternative explanation for the phenotype is based on the size of the different molecules.

The hydrodynamic radius of 10 kDa dextran is 20 Å, the diameter of the ConA tetramer is 80 Å, and two (or more) BSA molecules conjugated to a single 5 nm (50 Å) gold particle will have a diameter greater than 100 Å [73,74,75]. Thus, it seems that the small dextran molecules can access the FP while the larger proteins (whether lectins or trafficking in the fluid phase) cannot. This suggests that TbMORN1 may be involved in some kind of size-exclusion mechanism, potentially a kind of molecular valve to regulate flux into and out of the FP.

**Figure 4:**
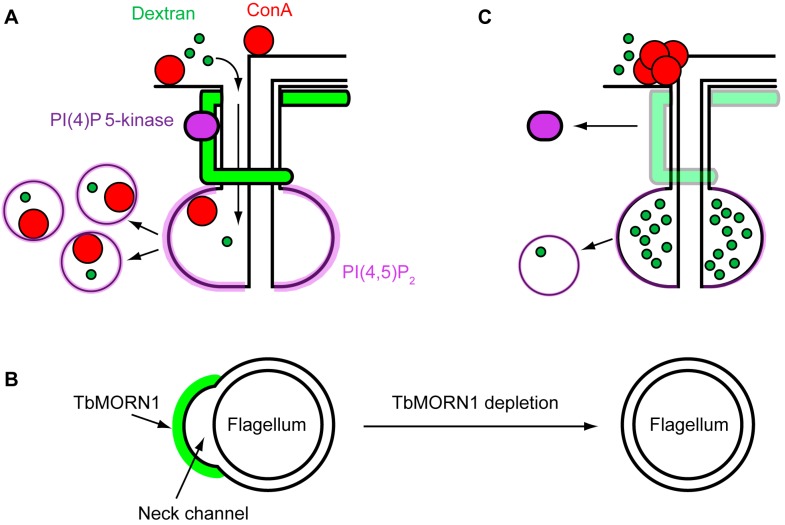
A hypothetical model for TbMORN1 function. (**A**) Schematic of possible function in wild-type cells. TbMORN1 (green) holds open the neck channel to facilitate macromolecule entry to the FP, and also acts as a scaffold for a PI(4)P 5-kinase. Material entering the FP is taken up in endocytic vesicles; (**B**) A transverse cross-section through the flagellar pocket neck. TbMORN1 molecules cluster on the cytoplasmic face of the membrane, causing it to bend inwards and create the neck channel. When TbMORN1 is depleted, the membrane is no longer bent outwards and the channel collapses; (**C**) The collapse of the neck channel inhibits the entry of large macromolecules such as ConA. Loss of TbMORN1 also impairs the localisation of the PI(4)P 5-kinase, resulting in a decrease in PI(4,5)P_2_ synthesis. The lower levels of PI(4,5)P_2_ on the FP compromise endocytosis.

Some tentative support for this hypothesis comes from morphological studies. As stated earlier, the distinguishing feature of the flagellar pocket neck is that the lipid membrane is tightly apposed with that of the flagellar membrane (Figure 1B). This tight apposition would seem to preclude the efficient entry of material into the FP. However, a small channel, formed by an inward furrow of the membrane, runs alongside the microtubule quartet and connects the FP lumen with the extracellular milieu [27]. Particles such as BSA-5 nm gold enter the FP through this “neck channel”. The possibility that the neck channel could be responsible for a size exclusion mechanism has already been noted in the literature [27]. Intriguingly, the position of TbMORN1 overlaps with the location of the neck channel (Figure 4A). TbMORN1 might therefore be responsible for holding the neck channel open. One hypothesis for how it could accomplish this derives from the fact that TbMORN1 is exclusively composed of MORN repeats. There have been persistent suggestions in the literature that MORN repeats can directly interact with lipids [76,77].

MORN repeats were first identified in junctophilins, proteins that link the sarcoplasmic reticulum to the plasma membrane (t-tubule) in skeletal muscle. Deletion of the MORN repeats seems to reduce plasma membrane association [35]. They are present also at the C-terminus of a family of plant PI(4)P 5-kinases [76,78,79,80]. It should be stressed that to date, however, there has been no really clinching evidence for a direct interaction with lipids. This shortfall is compounded by a frustrating lack of structural information on MORN repeats. Despite MORN-repeat proteins being found throughout the tree of life, only a single one is represented in the Protein Database of high-resolution three-dimensional structures [81,82,83]. There is consequently a clear need for more structural and biochemical information on this class of proteins.

A hypothesis for how TbMORN1 might hold the neck channel open would involve it physically bending the membrane of the flagellar pocket neck. It has been shown that lipid bilayers can be deformed solely through a protein crowding effect [84]. As such, a tightly-packed paracrystalline cytoskeletal structure might concentrate a large number of molecules onto the cytoplasmic face of the bilayer. Even if its components individually have only weak affinity for lipid, the resulting high-avidity interaction might be sufficient to bend the membrane inwards, creating a channel for the passage of macromolecules (Figure 4B). When TbMORN1 is depleted using RNAi, there are no longer sufficient molecules to bend the membrane, and the channel collapses (Figure 4B). Consequently, large molecules (such as ConA, 5 nm gold-BSA) can no longer get in (Figure 4C).

This hypothesis has appeal too in terms of cell biology. The surface of BSF cells is dominated by a single species of GPI-anchored variant surface glycoprotein, which naturally becomes a target for antibodies produced by the adaptive immune response. The BSF cells utilise hydrodynamic flow generated by their forward motility to sweep material towards the posterior pole of the cell [85]. Bound antibodies are thus swept into the FP, where they are internalised by endocytosis, routed to the lysosome, and degraded. The FP is then in some sense analogous to a plughole into which material for uptake is directed. This continuous movement of material towards the FP might explain why membrane-bound material that cannot enter the FP in TbMORN1-depleted cells (ConA) accumulates just outside it.

This does not explain why there is an apparent endocytosis defect following TbMORN1 depletion—dextran can get into the FP but does not appear to be taken up in quantity. An explanation for this effect might lie with lipid kinases. It has been noted already that in plants there exists a family of PI(4)P 5-kinases with MORN repeats [76,78,79,80]. These enzymes generate PI(4,5)P_2_, an essential cofactor for endocytosis. A recent study of PI(4)P 5-kinases in *T. brucei* identified an isoform (TbPIPKA) that appears to be present on the flagellar pocket neck [86]. Labelling of PI(4,5)P_2_ showed that this lipid is present only on the FP membrane. This by itself is highly unusual, as PI(4,5)P_2_ is normally found throughout the plasma membrane [87]. This spatial restriction of PI(4,5)P_2_ might reflect the fact that in *T. brucei*, endocytosis occurs only on this membrane.

The presence of a PI(4)P 5-kinase in the flagellar pocket neck suggests a model in which TbMORN1 may be a platform for lipid-modifying enzymes. In effect, this would mean that whereas the plant lipid kinases are localised by means of their *N*-terminal MORN repeats, in trypanosomes the same two protein modules (MORN repeats, kinase domain) are present on separate proteins. This enzyme platform would organise the seeding of the membrane in that subdomain with endocytosis-competent lipid. This would guarantee that only material swept into the FP can be taken up by endocytosis (Figure 4A). Depletion of TbMORN1 would mean that the platform for these enzymes is no longer there, causing them to mislocalise. The subsequent reduced production of PI(4,5)P_2_ in the flagellar pocket neck would result in a local concentration of the lipid that is now insufficient to support normal rate of endocytosis, leading to a reduction in uptake (Figure 4C). This hypothesis is by no means mutually exclusive with the idea of TbMORN1 also holding the neck channel open.

Membrane bending, platforms for lipid modifying enzymes, and an impact on endocytosis might seem far-fetched, but there are intriguing parallels in all of these concepts with structures in fungi called eisosomes [88,89,90]. Eisosomes (from the Greek “eis” meaning “portal” or “into”, and soma “body”) were first thought to be sites of endocytosis [91]. However, they are now seen as a distinct plasma membrane subdomain with a cytoskeletal patch positioned on the cytoplasmic side. Like the neck channel, they consist of a trench-like invagination of the plasma membrane [92]. Their exact function is still being elucidated, but they too have been associated with lipid-modifying enzymes, and are now recognised as being adjacent to sites of endocytosis [93,94,95,96]. There has been a concerted attempt to link eisosome concept with caveolae in mammalian cells, though this relationship seems strained [89,97]. There thus may be intriguing parallels between trypanosomes (Excavata) and yeast (Opisthokonta) in terms of plasma membrane subdomain organisation.

## 5. Conclusions

TbMORN1 is present in the hook complex, a multiprotein structure that is tightly associated with the single flagellum of the parasite *T. brucei.* The form (morphology), fabric (composition) and function of the hook complex have been the subject of in-depth inquiry through studies of TbMORN1, although a number of open questions remain. The current functional data suggest a possible link between TbMORN1 and a size-exclusion mechanism for entry into the FP, the sole site of endo- and exocytosis in the cell. Further investigation of this hypothesis and some of the other questions posed here, as well as characterisation of the cellular functions of other hook complex components, are likely to be fruitful areas of future research.
